# Integrating artificial intelligence into health care through data access: can the GDPR act as a beacon for policymakers?

**DOI:** 10.1093/jlb/lsz013

**Published:** 2019-09-16

**Authors:** Mélanie Bourassa Forcier, Hortense Gallois, Siobhan Mullan, Yann Joly

**Affiliations:** 1 Health Law and Policy Programs, Université de Sherbrooke, Sherbrooke, QC, Canada; 2 Center for Genomics and Policy (CGP), McGill University, Montreal, QC, Canada; 3 Université de Montréal, Montreal, QC, Canada; 4 Université Lille 2, Lille, France; 5 University of Sherbrooke, Sherbrooke, QC, Canada; 6 Department of Human Genetics Cross-Appointed at the Bioethics Unit, McGill University, Montreal, QC, Canada

**Keywords:** artificial intelligence, data protection, General Data Protection Regulation (GDPR), health care, Health Insurance Portability and Accountability Act (HIPAA), privacy

## INTRODUCTION

The potential of artificial intelligence (AI) to promote better health care has taken the centre stage in modern debates on public health and health policy. Although AI is considered a contemporary innovation, it has been in development for more than a half century. AI research began in the 1950s, when Alan Turing raised the idea that machines could 1 day think as humans.^1^ Then came, in 1959, the first instance of ‘machine learning’ (ML), where computer scientists created a program capable of solving puzzles on its own.^2^ Now, AI promises to lead the next major technological revolution, similar in stature to electricity and the internet.^3^

In the field of health care, AI has already led to improvements, particularly in areas such as precision medicine, diagnosis tools, psychological support, and help for the elderly.^4^ AI technologies generally require large amounts of both personal and non-personal data to function. In health care specifically, AI technologies rely on personal information, including health-related data extracted from medical files or research participants’ results.^5^ Promoting AI and capturing its benefits for the health care system yet depend, in large part, on procuring a convenient access to this sensitive data.^6^ Ensuring that privacy protections are in place appears essential, especially with individuals showing substantial concerns about sharing their data in the medical and clinical context.^7^

Suggestions to implement public open databases to promote medical research have created some controversy in Europe and in North America. In the UK, citizens rejected the care.data project launched in 2014 due to privacy concerns. Although widely supported by health care professionals, the failure of the project was largely due to a lack of transparency about the envisioned uses of health information and the possibility to opt-out.^8^ In the United States (US), studies have shown that individuals’ willingness to participate in research involving their genetic data is affected by their concerns about their ability to protect their privacy in such context.^9^ Paradoxically, this lack of trust is counterbalanced by a growing popularity of direct-to-consumer genetic testing and health monitoring devices. These devices create massive flows of personal and health data, mostly to private companies. This ambivalent attitude of individuals toward data sharing is a major issue for any privacy and data protection regulation. Adequately assuring the right to privacy of citizens while facilitating access to personal data for research is probably one of the biggest challenges policymakers have to face in any country wishing to benefit from many opportunities of AI technologies in health care.

Through the adoption of its new General Data Protection Regulation (GDPR),^10^ the European Union (EU) was the first to attempt to regulate AI through data protection legislation. This regulation paves the way for meaningful reforms in privacy legislation in the US and Canada. The GDPR covers all personal data processed by a data processor or controller established within the Union (art. 3.1, GDPR). It also extends to personal data of any data subject in the EU, no matter the establishment of the processor in two situations: whenever this processing is related the offering of goods or services to data subjects in the Union; and when related to the monitoring of their behavior within the Union (art. 2.2, GDPR). This means that many foreign companies’ activities may fall into the scope of the GDPR. Moreover, the extra-territorial reach of the new regulation puts pressure on Canada and the US to reform their own privacy legislation. Indeed, both systems likely fall short of some of the new requirements set by the EU regulation. If these laws are found to provide insufficient protection, the result could be a decrease in data flow from the EU to North America, due to the need to proceed via the adoption of additional contractual clauses.^11^ Such decrease would not only negatively affect research and development of AI technologies in both countries but would also interfere with any attempt at cooperation in the field.^12^ In this context, it appears all the more pressing to consider appropriate measures to consolidate privacy protection and promote stakeholders’ trust. After a brief overview of the contributions and promises of AI to the health sector, we will investigate the challenges to data and privacy protection brought about by developments in this field. This will lead us to identify key avenues for policy reform, in the US and Canada, which we contend could be inspired by the GDPR.

### Integrating AI: opportunities for health care systems and increasing need to access data

There is no single definition for AI. According to the Canadian Standing Senate Committee on Social Affairs, Science and Technology, the expression refers to ‘the reproduction of human cognitive functions such as problem solving, reasoning, understanding, recognition, etc. by artificial means, specifically by computer’.^13^ In many health care systems, AI has already been successfully deployed, mainly in the form of ML- and deep learning (DL)-based technologies.^14^ In both ML and DL, a certain amount of data (the input) is provided to the system for processing (through one or several algorithms), in order to provide an output. ML is more specifically used for automatic detection of patterns in large amounts of data, based on logical deduction. The key differences between ML and DL include the type and amount of data that can be processed by the system and how the algorithms are generated. DL describes a more complicated form of ML known as artificial neural network. In DL, the system can process larger amounts of raw and complex data, while ML is limited to a smaller amount of information which needs to be translated in a language that the machine is able to understand (referred to as ‘structured data’). The promise of DL has placed it at the heart of the debates around AI, especially in health care. The main evolution brought by the transition from ML to DL is that, while ML requires ‘human-built’, supervised algorithms, the process used in DL to obtain the output is generated by the system itself. The high level of complexity of the process also makes DL systems rather opaque. Concretely, programmers still know what is entered into the system and what comes out of it. However, it is nearly impossible to understand how exactly this output has been produced, even less to control it.^15^ In some cases, the data can become part of the algorithm itself. Some authors refer to this disturbing lack of transparency in DL as a ‘black box’ phenomenon,^16^ especially problematic when dealing with patients’ sensitive data.^17^ AI research applied to health care is a rapidly growing field.^18^ As of today, AI in health care is generally concentrated around three areas: oncology, neurology, and cardiology.^19^ These are all areas of medicine in which early detection is crucial.^20^ In clinical care, AI ML and DL systems are already assisting physicians in decision-making, providing them with relevant and up-to-date information for diagnosis and treatments. The use of AI, combined with imaging, has shown great potential in supporting the rapid identification of the presence or absence of certain types of cancer, sometimes with greater accuracy than specialists.^21^ DL systems have also shown great potential in promoting the development of precision medicine,^22^ using improved prognostic and diagnostic models.^23^ Electronic Health Records and telemedicine are also becoming widespread, showing significant capacity to shorten the time spent by health care professionals in addressing specific tasks.^24^ The use of carebots and other similar robots is growing in countries with acute aging populations.^25^

AI in health care is not limited to assisting in clinical care or decision-making. By automatically spotting similarities in patients’ medical records, AI systems can support researchers in quickly identifying the optimal patient cohort for a specific clinical trial.^26^ The ability of AI systems to make predictions based on larger sets of data can also benefit public health. AI based on Big Data has contributed to the development of ‘Precision Public Health’ to help predict and understand public health risks and customize treatments for definite and homogeneous subpopulations.^27^ In order to maximize these possibilities, considerable suggestions to transform health care systems by opening access to data collected in both clinical trials and medical care have garnered attention.^28^ The paradigm of a ‘Learning Health care System’ (LHS), for instance, describes a health care system ‘in which knowledge generation is so embedded into the core of the practice of medicine that it is a natural outgrowth and product of the health care delivery process and leads to continual improvement in care’.^29^ A LHS is thus based on the integration of research and practice as a way of facilitating data and knowledge transfers by improving access to medical data (collected in electronic health files for example). The blurring of the boundary between research and practice entailed by the LHS does conflict, to some extent, with traditional legal and ethical norms. These norms were built around the belief that research and clinical care need to be clearly delineated to protect patients and research participants.^30^ The LHS paradigm requires novel, more appropriate, ethical and regulatory frameworks^31^ which should integrate privacy and data protection mechanisms, as those are a crucial vector of success of this enterprise.

### Implications for privacy and data protection

#### Implications for privacy

Privacy can be understood as a person’s right to control access to her personal information. This relates to information that a person desires to keep for herself, or only wishes to share with a small group of people such as health data, religious belief, political opinions, and sexual preferences. The unprecedented challenges the right to privacy faces with the development of the Internet and AI was unanticipated at the time modern privacy laws were developed. ^32^ The root of the differences between the American, Canadian, and European frameworks on data protection can be found in their respective conceptual foundations for protecting privacy. In the US and most of English Canada, privacy protection is grounded in the protection of liberty, especially freedom from government intrusions. The US legal framework on privacy is a complex, sometimes conflicting, patchwork of federal and state laws, and sector-specific regulations.^33^ In contrast, Europe and Canada have adopted a more uniform, comprehensive, approach to privacy and data protection. In Europe and in Canada, especially in the province of Quebec, privacy is conceived as an essential component of the right to human dignity. For some, the Canadian conception of privacy is a ‘middle ground between the EU and the US, as Canadians share Americans’ concerns about government intrusions, while also having deep worries about private sector abuses of their personal information’.^34^ Privacy is established as a fundamental Human right in European law (Article 8, ECHR) since 1953. It benefits from a similar recognition in the Quebec Charter (Article 5, CHRF, QC). However, in the US and English Canada, privacy is not explicitly included in the Constitution as its protection rather derives from jurisprudential interpretation.^35^ Beyond these conceptual differences, individuals on both sides of the Atlantic have increasingly shown a paradoxical attitude toward personal privacy. While sometimes reluctant to allow access to data for health research, most willingly share personal data through portable devices and consumer genetic testing websites on a regular basis. ^36^ This paradoxical behavior is concerning, since health data is often logically surmised from other personal data provided online, such as consumption habits.^37^ This phenomenon creates important challenges for privacy protection and regulations. One obvious example was given by the illegal use of personal data compiled by Facebook to support the 2016 American presidential campaign.^38^ Such events, highly covered in the media, have drawn the attention of the public to the risk of personal data usage for commercial or political purposes without proper consent. They also highlight the lack of adapted privacy and governance frameworks to prevent unwanted uses of personal data, especially on the Internet.

Secondary uses (and misuses) of data are an issue that the European legislators wished to address when adopting the GDPR. Applied to the medical realm, the GDPR has a dual purpose. On the one hand, it aims at strictly preventing unconsented and secondary uses of personal data (both by the private and public sector). On the other, it aims at streamlining access to personal data, increasingly necessary for the development of research, while remaining mindful of the importance of privacy. To that extent, the GDPR provides for a reduction of the obligations in terms of administrative formalities before accessing and using health data. Where heavy declaration formalities to national authorities were in place under the Directive, the GDPR aims at making data actors more accountable rather than restricting their ability to start research in the first place.^39^ Health-related data, more specifically, are categorized as ‘sensitive data’. These data are protected by a specific framework which prohibits their processing (art. 9, GDPR). Substantial exceptions are yet provided in order to facilitate access to relevant data while acknowledging their sensitivity.

#### Implications for data protection

Although sometimes presented as separate rights in legal systems, the right to data protection is an essential component of the right to privacy. Consequently, where data protection cannot be guaranteed, the respect of privacy is equally impossible to ensure. In Canada and in the US, data protection regulations cover the collection and the use of personal data. Sometimes, however, AI is not based on any personal data, meaning that no data protection regulation applies in these circumstances. The criteria for defining what is personal versus non-personal data then become crucial for determining the scope of application of a data regulation. The EU has, however, adopted a specific regulation for non-personal data. This regulation aims at strengthening the free circulation of non-personal data and facilitating the development of a common digital market within the EU.^40^

Generally, personal data are data that allows the direct or indirect (eg through triangulation) identification of a data subject.^41^ Canadian federal law defines personal information as ‘information about an identifiable individual’ (art. 2, PIPEDA). This definition is similar to that embodied in the GDPR which covers ‘any information relating to an identified or identifiable natural person (data subject)’ (art. 4, GDPR). In the US, there are multiple definitions of personal data in the legislation, as applicable regulations vary according to sectors and states.^42^ Simply put, all three considered jurisdictions appear to share significant similarities on what is considered personal data. However, differences do exist affecting the way privacy is actually protected. In particular, the new European regulation goes beyond the general classification of personal versus non-personal data. Data concerning health, genetic data, and biometric data, in particular, are considered highly sensitive. ‘Sensitive data’ are assigned a more protective framework by the GDPR than that applicable to other types of personal data (art. 6, GDPR) mentioned, the processing of all sensitive data is prohibited under the GDPR but Article 9.2 provides with a substantial list of exceptions to this general prohibition principle. The first of these exceptions applies where ‘the data subject has given explicit consent to the processing of those personal data for one or more specified purposes’ (art. 9.2.a, GDPR). Interestingly, these exceptions are established as alternative conditions. The wording of the article thus implies that obtaining specific, informed consent as required under the GDPR consent is not necessary, as long as another legal basis for the processing applies. For example, the processing of sensitive data is allowed when ‘necessary for archiving purposes in the public interest, scientific or historical research purposes or statistical’ (art. 9.2.j, GDPR), provided appropriate safeguards are in place (art. 89, GDPR). We understand that the aim of the GDPR is to allow some flexibility in the context of scientific research using sensitive data. Member states, however, ‘may maintain or introduce further conditions, including limitations, with regard to the processing of genetic data, biometric data or data concerning health’ (art. 9.4 and 89, GDPR), and harden consent requirements with ‘specific provisions’ (art. 6.2 and 9.2.a, GDPR). Such discretion could, in our view, hinder this objective and has been criticized for its damaging impact on international harmonization initiatives.^43^

Interestingly, in the US, a federal regulation also covers Protected Health Information (PHI) collected by defined entities as a specific type of data.^44^ The *Health Insurance Portability and Accountability Act* (HIPAA)^45^ provides with specific standards for the collection and the use of PHI since 1996. However, the efficacy of HIPAA to adequately protect health data and patient’s privacy is greatly challenged by its restrictive scope of application. HIPAA only applies to data processed by ‘covered entities’ and ‘business associates’, meaning that data miners are typically excluded from its application.^46^ Technology giants like Google, Amazon, which deal daily with large amounts of personal data including health-related ones, are generally not covered by the regulation.^47^ Moreover, HIPAA’s ‘Privacy Rule’ only protects identifiable information (part 160 & 164). Under American law, any ‘de-identified’ information is considered non-personal, meaning that it is not subject to any data protection regulation. Under HIPAA, de-identification can be completed in two ways: either by removing specific identifiers enumerated in the law from the data set or by having a statistical expert confirm that the risk of re-identification linked to a specific data set is sufficiently small (45 CFR § 164.514(b)(1), HIPAA). Both de-identification techniques have been proven insufficient to prevent all re-identification of data subjects.^48^ The HIPAA de-identification standard has thus been considered particularly problematic in the case of genomic and genetic data. This type of data is commonly treated as de-identified as soon as all personal information related to the dataset is removed (ie name, age, address, etc.) and falls out of HIPAA’s scope of application. This occurs even though the actual de-identification of genetic information is unlikely and despite the fact that this type of data can be sensitive.^49^ In this case, other laws at the federal (the Genetic Information Non-discrimination Act, for one) or state level can sometime offer additional protection.

Under the GDPR, de-identification is not automatically considered a sufficient way to prevent re-identification of individuals. As such, de-identified data remain in the category of protected personal data. The GDPR only excludes anonymous data from its scope of application. Strict conditions are set around anonymization under the GDPR. The process of anonymization involves a number of techniques designed specifically to prevent re-identification. Recital 26 specifies, regarding these techniques, that:

‘To determine whether a natural person is identifiable, account should be taken of all the means reasonably likely to be used, such as singling out, either by the controller or by another person to identify the natural person directly or indirectly. To ascertain whether means are reasonably likely to be used to identify the natural person, account should be taken of all objective factors, such as the costs of and the amount of time required for identification, taking into consideration the available technology at the time of the processing and technological developments.’

The categorization of sensitive data presents advantages, as it accounts for the need for additional caution when dealing with health-related data. It can be efficient to prevent unconsented secondary uses, but this special category can also act as a disincentive for researchers, especially given the high sanctions they incur in case of sensitive data breach.

Such legal distinction between personal data and sensitive data does not exist in the Canadian regulation on data protection. At the federal level, data protection is covered by two main regulatory instruments, the Personal Information Protection and Electronic Documents Act (PIPEDA),^50^ which applies to the private sector, and the Privacy Act, which covers federal agencies. Several provinces have adopted their own regulations pertaining to health information, considered to be substantially similar.^51^ PIPEDA and its provincial equivalents apply to private organizations which collect, use or disclose personal data for commercial purposes. In that sense, their scope of application is more inclusive than HIPAA’s, as they encompass any processing of personal data performed by a company (art. 2, PIPEDA). Under the previous Directive on data protection, PIPEDA was considered adequate to protect personal data covered by EU law.^52^ Adequacy is especially important to guarantee data flows from the EU to Canadian companies. Article 46.5 of the GDPR allows PIPEDA to benefit from this beneficial status until the next evaluation. The lack of specific protection mechanisms, including regarding sensitive data, could yet impede PIPEDA from maintaining adequacy. Without such status, each international personal data transfer is only allowed by exception. Such exceptions include when the data subject has explicitly consented, after being informed of the absence of an adequacy decision and the risks associated (art. 49.1.a, GDPR). Other possibilities include when a transfer is necessary for the performance of a contract with the data subject, or for ‘important reasons of public interest’ (art. 49.1.b and d). Such requirements can be impractical, especially in scenarios where large amounts of data are needed to develop a DL system, for example.

PIPEDA has yet to be updated to take into consideration the implications of AI on privacy and data protection, especially in case of sensitive and health-related information. In an op-ed following the revelations of illicit uses by Facebook of personal data for political purposes, the Privacy Commissioner of Canada warned against the limitations in Canada’s legislation on privacy and urged for the revision of PIPEDA.^53^ In line with the recommendations formulated by the Privacy Commissioner, the Committee on Access to Information, Privacy and Ethics of the House of Commons published a report with comparable reform propositions for PIPEDA. The Committee emphasizes on the necessity to adapt Canada’s legislation in order to maintain adequacy status with European regulations and prevent a potential chilling effect on commercial exchanges with the EU.^54^ Meanwhile, the Council of Canadian Academies concluded that Canada’s current legal framework on data regulation is not only unsuccessful in protecting privacy but also gravely hinders timely access to data for health research. The reporters recalled the dilemma policymakers are faced with and the growing need for amendments to solve it: ^55^

‘The primary, overarching challenge in Canada, as in other jurisdictions, is to meet two fundamental goals at the same time: to enable access to health and health-related data for research that is in the public interest, on the one hand, and to respect Canadians’ privacy and maintain confidentiality of their information when it is used for research, on the other.’

In order to simultaneously better protect privacy, prevent unconsented uses of data and favor research through data sharing, both the Canadian and the American frameworks are faced with the need for substantial revisions. Such amendments should, in our view, aim at reinforcing individuals’ rights over their own data. The new consent requirements as well as innovative rights implemented in the GDPR are designed to enable individuals to become more proactive in the protection of their privacy, providing interesting basis of inspiration for regulators. Meanwhile, the GDPR also provides reinforced obligations for data actors, which need to be considered, both in the American and in the Canadian contexts.

### Improving privacy and data protection in Canada and the US: insights from the GDPR

#### Reinforcing rights: toward better control of data owners on their personal information

##### Right to consent and Automated processing

In order to grant individuals with higher control over their data, the GDPR strengthens the requirements for valid consent set forth by article 7. Valid consent is now defined as ‘freely given, specific, informed and unambiguous’ (art. 4.11, GDPR). Although the GDPR does not state that consent must be written, it should be explicit and informed. Recital 32 specifies on this point that ‘silence, pre-ticked boxes or inactivity should not therefore constitute consent’. More concrete steps for assessing what constitutes explicit valid consent under the GDPR yet remains open to interpretation by national jurisdictions.^56^ This margin of discretion is meant to provide courts of justice sufficient flexibility when evaluating the validity of consent models. These new requirements promote the use of more meaningful consent processes over rigid bureaucratic procedures. They are meant to foster the level of trust necessary for a more optimal data sharing, primarily by increasing individuals’ sense of control over their own data. However, if consent requirements are more stringent, many exceptions are set up by the GDPR which allow the processing of both sensitive and non-sensitive data. To be lawful, any processing requires a lawful basis, and valid consent is only one of the possible bases listed in article 6 for the use of non-sensitive data. The five other bases include ‘when the processing of personal data is necessary for the performance of a contract to which the data subject is party’ or ‘a task carried out in the public interest’ (art. 6.b and 6.e, GDPR). Consequently, as long as any other basis can apply, the lawfulness of the process is no longer conditioned by the obtaining of a valid consent.

In the case of sensitive data, article 9 states that processing based on consent should be limited to pre-defined purposes and any further processing of the same data implies that consent is again sought. However, secondary uses of data are allowed when such processing seeks scientific research purposes (art. 89.1, GDPR). Allowing the use of the same dataset for several purposes is thought as beneficial, if not crucial, to most scientific development.^57^ Recital 156 specifies however that:

‘The further processing of personal data for archiving purposes in the public interest, scientific or historical research purposes or statistical purposes is to be carried out when the controller has assessed the feasibility to fulfill those purposes by processing data which do not permit or no longer permit the identification of data subjects, provided that appropriate safeguards exist’.

Secondary uses of data without the previous obtaining of new consent from data subjects are thus exceptionally admitted under the GDPR, but constrained by strict conditions that may ultimately discourage researchers from taking full advantage of the GDPR’s exception. The new consent requirements do offer interesting perspectives of evolution for both the American and the Canadian frameworks which could help generate trust among data subjects.

In both the Canadian and the American frameworks, valid consent is generally required for the use and processing of personal data. However, in both frameworks, what constitutes valid consent is not always in line with the GDPR’s new dispositions, especially as to the point of explicitness. These differences in valid consent requirements between the new European regulation and both North American laws could mean that a data process considered lawful under PIPEDA and/or HIPAA may be found unlawful under European law. Specifically, the GDPR intends to prevent implied consent scenario, such as ‘opt-out’-based participation, for example, as it is not always clear to data subjects what they are actually consenting to. A major challenge for any company dealing with personal data covered by the GDPR is thus to ensure that consent is duly informed and expressed. These new consent requirements have already led to the imposition of a record fine of €50 million—about US$75 million—for the tech giant Google by the French privacy national authority. Among other breaches, the French authority condemned the American company for establishing consent requirements which left users ‘unable to understand the extent of the treatments implemented by Google’.^58^ This case, thought to be the first of several to come, demonstrates the problematic gap between the new GDPR provisions and the North American laws.

If modifications of consent requirements are needed in both frameworks to better protect individuals’ privacy and interests, these should consider the specificities of scientific research, in order not to slow down the development of useful technologies. Specific consent requirements, limited to pre-determined purposes, can be quite limiting in the research context, as it is often difficult to foresee the potentialities of the data collected. Recital 33 of the GDPR acknowledges this challenge, yet does not provide a means to avoid the obligations outlined in the GDPR. ^59^ The Working Party on the Protection of Individuals with Regard to the Processing of Personal Data, addressed this issue, in stating that, in situations where informed consent may not be obtained, other methods could be used. For instance, it would be possible to define the research in the earlier stages in more general terms consent may be obtained at later stages, as the research advances.^60^ The Working Party admitted another form of consent to apply in some instances, known as dynamic consent. Dynamic consent is a model based on modern communication strategies to ensure that subjects are continuously and appropriately informed about the different uses made of their data. Although appealing, the practical constraints brought by a dynamic consent approach can be burdensome for researchers seeking to implement this model on a large scale. Another alternative could be the generalization of broad consent to favor research, which means consent given to a well-defined framework for future research of certain types, without the need to seek for consent any time a utilization is considered. In a broad consent framework, informed consent is not required for each specific purpose, but a substantial change in the framework would oblige a researcher to re-consent the research participants. To ensure privacy protection, such framework should include an ethical review of each distinct research project, as well as strategies of robust self-regulation. Making broad consent the rule and specific consent the exception in scientific and health care research could be an interesting alternative, especially in fields like biobanking, where the re-usability of biological samples and data are central.^61^ Consent requirements should be reinforced in the American and in the Canadian frameworks, but in research, and especially in health care, broad consent seems well suited to the biobanking context and potential AI technologies based on the data gathered.

The GDPR provides data subjects with another right that aims at reinforcing their sense of control over the proceedings of their data. This right affirms that they will ‘not to be subject to a decision based solely on automated processing, including profiling’ (art. 22, GDPR). AI technologies in health care may be directly concerned by this new right. This right implies, for example, that a DL system created to provide treatment suggestion cannot be used as the sole basis for deciding which drug will eventually be prescribed. Exceptions to this rule do apply when provided by Member States’ law or when necessary to enter into a contract or when the processing is based on the individual’s prior consent. Data subjects have the right, in such cases, to receive a justification for the automated decision. Yet an issue arises when AI becomes so complex and processes such voluminous amount of data that a justification cannot be given. The “black-box” phenomenon of DL systems can then become a real hurdle for the implementation of AI in health care.

##### Right to data portability and Right to be forgotten

Personal information under the GDPR is to be obtained in a structured, commonly used and machine-readable format (art. 20). In all three contexts under study, data subjects also have a general right to access and correct the personal data that an organization has collected on them. The GDPR includes an interesting new right to data portability. This right aims at enabling individuals to take better control over their data and encourage competitiveness.^62^ In health care, data portability is thought to have practical benefits for data access, as it enables users of fitness trackers, for example, to save years of data compiled on an app and share it with their physician or with a research endeavor they wish to support.^63^ Yet, Article 20 excludes personal data which has not been provided by the data subject herself from its scope of application. The data resulting from the analysis made of the data collected by a health wearable device, for example, is thus not covered by this right, despite its potential benefit further research.^64^

Under HIPAA, individuals have a comparable right to get their information transferred from one health service provider to another. Data portability in the US context can, however, lead to a weakened protection of health data, as personal health information (PHI) is only protected when held by ‘covered entities’, for example, health plans, health care clearinghouses, and any health care provider. As previously mentioned, although seemingly large, this category leaves out substantial areas. It excludes public health agencies, law enforcement agencies or personal health record vendors, for example, meaning that any PHI held by a non-covered entity is no longer under HIPAA’s protection. A health record transferred to a non-covered entity may then, at best, fall into the scope of application of another privacy regulation, but could also be completely passed over by US data regulation.^65^ While data portability can be beneficial for research by facilitating data transfers, in the US context, it also reduces HIPAA’s already limited scope of application.

In Canada, PIPEDA contains no obligation regarding data portability. Given its potential to favor data transfers in research (by facilitating data transfer from any file to a research project in which a data subject wishes to participate, for example), the Committee on Access to Information, Privacy and Ethics recommended that PIPEDA be amended ‘to provide for a right to data portability’.^66^ The right to data portability should be seen as a quintessential part of any privacy regulation at the time of AI, as it empowers individuals to better control the use of their data by re-directing it where it is most useful. But, it can only do so if comprised in an adequate and all-encompassing health data protection framework, in order to ensure that sensitive data transfer will not lead to a deregulation of this data.

Another novel right granted under the GDPR to data subjects is the right to erasure (art. 17, GDPR). The right to erasure or right to be forgotten enables individuals to require complete deletion of their personal data held by either private or public organizations. It is a GDPR novelty which is absent from both HIPAA and PIPEDA. This right is meant to assure data subjects that any personal information they disclose can be completely erased at their request. Article17 also provides some implicit restrictions to the application of the right to erasure, as it only applies when based on one of the legal grounds enumerated. These grounds include personal data collected which is no longer necessary in relation to the purposes for which they were collected in the first place. Should this data still be necessary for the processing and no other legal ground apply, the request for erasure could be validly denied. Other exceptions include when the processing is necessary for reasons of public interest in the area of public health (art. 17.1.c, GDPR) or when it involves the exercise of official authority (art. 17.1.d, GDPR). In cases where the right to erasure does apply, its implementation will be hindered by the technical difficulty to ensure complete and systematic deletion of personal information, especially when already shared with collaborators.^67^ In the context of AI, one specificity of DL is that the algorithm used to obtain an output is automatically created based on data that has been previously introduced. This data thus becomes part of the algorithm and it becomes impossible to identify and extract specific data in order to erase it. The GDPR’s high sanctions linked to the violation of its provisions could apply in case of failure to erase. This is part of a general intention of the EU legislator to increase the accountability of data actors for protecting privacy and to promote the development of self-regulation to avoid situations of non-compliance. In the health care field, the right to erasure implies that health care providers may be forced to delete medical records at their patients’ request. Consequently, the integrity of the information held in a patient’s medical record could become difficult to preserve, which might adversely affect health care. Health care providers are already expressing concerns related to ‘information blocking’ problem when accessing patients’ Electronic Health Records. Such blockages often result from technical factors like incompatibility between two record systems, but also from stringent privacy-related regulations. Access to complete clinical information at the point of care is yet crucial.^68^ The Canadian and American legislators may want to keep this in mind if they decide to implement a right to erasure in their jurisdiction. They should at least guarantee that such right does not become too burdensome for health care structures trying to implement helpful technologies, by limiting its scope of application for example.

#### Reinforcing obligations: toward increased accountability of data actors

##### Data protection assessment, privacy by design, and privacy by default

The GDPR places a greater emphasis than its Canadian and American counterparts do on transparency, fairness, and accountability (art. 5, GDPR). To favor a preventive approach toward the protection of privacy and personal data, the GDPR encourages the adoption of technical means prior to processing personal data (art. 25 and rec. 78, GDPR). The GDPR also requires that a data protection impact assessment (DPIA) be made whenever a data process ‘is likely to result in a high risk to the rights and freedoms of natural persons’ (art. 35, GDPR). Although examples of such data process are provided (art. 35.3, GDPR), the wording of the text suggests that this list is non-exhaustive. Accordingly, the article 29 Working Party issued Guidelines to help determine when a DPIA is required.^69^ Among other criteria, a processing should be considered ‘likely to result in high risk when it involves sensitive data, data concerning vulnerable subjects (eg patients), or when it is based a new technology or an innovative use of an existing one. Since applications of AI in health care are usually based on health-related data and often constitute technological novelties, a DPIA is ultimately likely to be required before any processing in the field. ^70^ At a minimum, a DPIA must address: (i) a description of the processing operations and purpose of processing; (ii) an assessment of the need for and proportionality of the processing, and the risks to data subjects (viewed from the perspective of the data subject); and (iii) a list of the measures to mitigate those risks and ensure compliance with the GDPR. An exception to the requirement to perform a DPIA can be found at recital 91, which specifically provides that:

‘the processing of personal data should not be considered to be on a large scale if the processing concerns personal data from patients or clients by an individual physician, other health care professional or lawyer. In such cases, a data protection impact assessment should not be mandatory’.

Although absent from PIPEDA, the DPIA mechanism is mandatory under Canadian law for public federal government institutions only. Under US law, HIPAA also recommends performing a risk assessment annually to ensure that entities are compliant to HIPAA’s requirements. This assessment is, however, different from the GDPR’s DPIA, which is conceived as a continuous and preventive process, undertaken before the processing. The DPIA is, in that sense, quite unique to the EU regulation. Moreover, under the GDPR, data actors are required to ‘integrate the necessary safeguards into the processing in order to meet the requirements of this regulation and protect the rights of data subjects’ (art. 25, GDPR). This principle, known as ‘privacy by design’, was actually developed in the 1990s in Canada by Ontario’s former Privacy Commissioner Ann Cavoukian.^71^ Curiously, it has not been formally integrated in the Canadian PIPEDA. Accordingly, the Privacy Committee of the House of Commons commended in 2018 that ‘PIPEDA be amended to make privacy by design a central principle and to include the seven foundational principles of this concept’.^72^ The GDPR has also enforced a new ‘privacy by default’ principle (art. 25, GDPR). This principle involves that the technical means for data minimization be implemented when developing a technology. Data processors are now obligated to set up technical guarantees ensuring that only the data actually necessary for the completion of predefined finalities be accessed and processed. By imposing the implementation of preventive mechanisms such as privacy by design and by default, the GDPR encourages the rapid adoption of appropriate technical measures to prevent potential breaches. These new obligations for technology developers contribute to promote self-regulation and are assorted with significant sanctions.^73^ Including new requirements such as of privacy by design and by default in privacy law would substantially help in reducing the incidence of data breaches in the US and Canada.

##### Higher penalties and security breach notification requirements

Perhaps the most significant difference rising from the comparison of PIPEDA, HIPAA and the GDPR are linked to stances on penalties and security breach notifications.

As to security breach notifications, HIPAA’s Privacy Rule requires covered entities to notify individuals of any breach in protected health information security within 60 days (45 CFR §§ 164.400-414, HIPAA Breach Notification Rule). In Canada, provincial health privacy laws in Alberta, Ontario, New Brunswick, and Newfoundland and Labrador have also enforced data breach notification systems.^74^ Amendments to PIPEDA that entered into force in November 2018 include a new data breach notification obligation to the Privacy Commissioner. However, some have criticized the imprecise language of the new text, requiring notifications only in case of ‘real risk’ of ‘significant harm’.^75^

The US has also been the first to match privacy breaches with high penalties. Similar to the US model, the GDPR has set up heavy sanctions in case of privacy breach and data leakage. However, the US framework lacks a central data protection authority. Also, if sanctions and breach notification mechanisms do exist, the disparities in privacy and data protection between States and sectors makes them hard to compare with the new European standards.^76^ Under the GDPR, there are two levels of penalties. At the lower level, a data user may be fined up to €10 million—about US$11.3 million, or 2 per cent of the worldwide annual revenue of the prior financial year (art. 83, GDPR). At the upper level, a firm may be fined up to €20 million—about US$22.6 million, or 4 per cent of the worldwide annual revenue of the prior financial year (art. 83, GDPR). This dissuasive system aims at increasing the accountability of actors dealing with personal data and encouraging self-regulation to prevent privacy breaches. These higher sanctions come with significant administrative simplifications. Under the former Directive, heavy declaration formalities to authorities in charge of privacy at the national were mandatory prior to any collection or processing. In an attempt to alleviate administrative tasks for data processors, the GDPR no longer contains declaration requirement but now requests organizations to keep a record of their activities (art. 30, GDPR). This simplification comes at the cost of granting national privacy authorities with higher investigatory and sanction powers.

In Canada, PIPEDA is much more conservative with its fines.^77^ Since the enforcement of the Digital Privacy Act, failure to notify a data breach is sanctioned by fines of up to C$100,000—about US$75,300 (art 28, PIPEDA). However, the enforcement powers of the Privacy Commissioner are limited. As currently framed, the Privacy Commissioner has no authority to order changes or emit sanctions in case of non-compliance. At the provincial level, some privacy commissioners (ie Alberta and Quebec) have such authority.^78^ The lack of harmonization results in somewhat uneven practices on the Canadian territory. Following the Facebook data breaches, the opportunity to provide Commissioner with more extended investigation and sanction power may warrant further consideration.^79^ If sanctions under PIPEDA may be insufficient to ensure compliance from industrial giants, the US legislation does not provide a satisfying harmonized framework on the territory, making high sanctions difficult to apply at times.

## Conclusion and discussion

The opportunities brought by the integration of AI technologies in the field of health care should not be underestimated. AI developments can help sifting through huge volumes of data to detect patterns, correlations and perform complex calculations, tasks that machines are better equipped to perform than humans are. Many such applications are already in use, helping health care providers save time and money and improving health research and patient care. However, should individuals become reluctant to provide access to their personal data, the impact would be devastating for the implementation of AI in any health care system. A general loss of trust from the public is understandable given the high-profile examples of misuses of personal data such as revealed by the Cambridge Analytica case.^80^ The GDPR’s new mechanisms aiming to prevent such unwanted uses of personal data, especially through the prohibition of opt-out scenarios and some consent requirements, could guide North American policymakers. The specificities of AI and the novel risks brought for privacy protection are, at least partially, addressed in the GDPR: although some mechanisms seem restrictive and could be problematic for AI developers (such as the right to erasure), the general effort toward increased responsibility of data actors must be acknowledge and should inspire the adoption of more protective regulations.

We contend that the political and economic impetus to align data privacy law with the GDPR also calls for legal reform, in order to make the Canadian and US legislation more relevant to the unique privacy challenges raised by AI. While HIPAA provides protective mechanisms as well as substantive penalties, it only targets identifiable data collected by covered entities and business associates. Canada has an all-encompassing law that applies to all industries and all personal data collected for commercial purposes. However, stakeholders agree that it is outdated to respond to the reality of AI and the internet. The extra territoriality and expended duties imposed by the GDPR regulation maybe a source of discontent and uncertainty and may negatively affect data sharing for a time. This should not overshadow interesting novel features included in the EU regulation that would help better address the challenges posed by AI. Any legal reform should certainly consider these elements for Canada and US privacy legislations but should also keep in mind that the GDPR’s application scope is yet to be defined through case law. Any uncertainty regarding the GDPR’s potential application could be addressed in the reforms to come in North America. In fact, Health Canada, which is revising its medical devices regulations, seems to be aware of this opportunity. It is anticipated that any medical device with automated data profiling will be approved if it comes with a ‘white box’, by opposition to the problematic ‘black box’. The white box should allow to understand the way the profiling is created by the device, limiting the risks associated to biased automated profiling.

Finally, we believe that developing a compatible international framework to protect personal information that enables responsible data sharing and cross-border data transfers would be beneficial to all parties.^81^ Let us also not forget that, ultimately, the benefits we can expect of AI are directly determined by the sum of data such technology is allowed to use and be trained on.

## Supplementary Material

Response_to_Decision_Letter_July_4_lsz013

## Footnotes

1 See Allan M. Turing, *Computing Machinery and Intelligence*, 49 Mind 433, 460 (1950); Nils J. Nilsson, *The Quest For Artificial Intelligence*, CambridgeCore 56 (2009), http://core/books/quest-for-artificial-intelligence/32C727961B24223BBB1B3511F44F343E (accessed Mar. 14, 2019).

2 See Nello Cristianini, *Intelligence Reinvented*, 232 NewScientist 37, 41 (2016).

3 See Roger Parloff, *The Deep-Learning Revolution*, 174 Fortune 96, 106 (2016).

4 See Andreas Holzinger, *Trends in Interactive Knowledge Discovery for Personalized Medicine: Cognitive Science meets Machine Learning*, 15 The IEEE IntelligentInformaticsBulletin 6, 14 (2014).

5 See Riccardo Miotto et al., *Deep Learning for Healthcare: Review, Opportunities and Challenges*, 19 BriefBioinform. 1236, 1246 (2018).

6 See Willem Sundblad, *Data is the Foundation for Artificial Intelligence and Machine Learning Forbes* (2018), https://www.forbes.com/sites/willemsundbladeurope/2018/10/18/data-is-the-foundation-for-artificial-intelligence-and-machine-learning/ (accessed Jan. 23, 2019).

7 See Brian Scogland, *Artificial Intelligence in Medicine: Hope or Hype? MDDI Online* (2018), https://www.mddionline.com/artificial-intelligence-medicine-hope-or-hype (accessed Jan. 9, 2019).

8 See Nick Triggle, *Care.data: How Did It Go So Wrong?* (Feb. 19, 2014), https://www.bbc.com/news/health-26259101 (accessed Jun. 14, 2019).

9 See Ellen W. Clayton et al., *A Systematic Literature Review of Individuals’ Perspectives on Privacy and Genetic Information in the United States*, 13 PlosOne 2 (2018).

10 Regulation (EU) 2016/679 of the European Parliament and of the Council of April 27, 2016 on the protection of natural persons with regard to the processing of personal data and on the free movement of such data, and repealing Directive 95/46/EC (General Data Protection Regulation). [2016] OJ L119/1.

11 See H. Evans and S. Togawa Mercer, *Privacy Shield on Shaky Ground: What’s Up With EU-U.S. Data Privacy Regulations*, Lawfare (2018), https://www.lawfareblog.com/privacy-shield-shaky-ground-whats-eu-us-data-privacy-regulations (accessed Feb. 7, 2019); S. K. Robertson, *Calls Grow for Canada to Modernize Privacy Laws Amid EU Changes*, TheGlobe andMail (2017), https://www.theglobeandmail.com/report-on-business/industry-news/marketing/calls-grow-for-canada-to-modernize-privacy-laws-amid-eu-changes/article35778176/ (accessed Feb. 7, 2019).

12 International collaboration in AI is based on increasingly large amounts of data, including trans-border data sets. If data exchanges are hindered, the continuation or development of transatlantic research projects in the field are thought to be impeded. See Mark Phillips, *International Data-Sharing Norms: From the OECD to the General Data Protection Regulation (GDPR)*, 137 Hum. Genet. 575, 582 (2018).

13 See K. K. Ogilvie and A. Eggleton, *Challenge Ahead: Integrating Robotics*, ArtificialIntelligence and 3D PrintingTechnologies intoCanada’s HealthcareSystems 5 (2017).

14 See F. Jiang et al., *Artificial Intelligence in Healthcare: Past, Present and Future*, 2 StrokeVasc. Neurol. 230, 243 (2017).

15 See W. Knight, *The Dark Secret At The Heart of* AI—MIT *Technology Review* (2017), https://www.technologyreview.com/s/604087/the-dark-secret-at-the-heart-of-ai/ (accessed Jan. 9, 2019).

16 See Roger A. Ford and W. Nicholson Price II, *Privacy and Accountability in Black-Box Medicine*, 23 Mich. Tech. L. Rev. 1 (2016); W. Nicholson Price II, *Regulating Black-Box Medicine*, 116 MichiganLawReview 421 (2017); T. Simonite, *AI Experts Want to End “Black Box” Algorithms in Government | WIRED*, Wired (2017), https://www.wired.com/story/ai-experts-want-to-end-black-box-algorithms-in-government/ (accessed Jan. 9, 2019).

17 About ‘black box’ technology, the AI Now Institute of NYU recommends that ‘*core public agencies, such as those responsible for criminal justice, healthcare, welfare, and education refrain from using “black box” AI and algorithmic systems*’ *id*. A. Campolo et al., AI Now 2017 Report (2017), https://assets.ctfassets.net/8wprhhvnpfc0/1A9c3ZTCZa2KEYM64Wsc2a/8636557c5fb14f2b74b2be64c3ce0c78/_AI_Now_Institute_2017_Report_.pdf (accessed Jan. 9, 2019).

18 See D. Faggella, *The State of AI Applications in Healthcare—An Overview of Trends*, Emerj (2018), https://emerj.com/ai-sector-overviews/state-ai-applications-healthcare-overview-trends/ (accessed Jan. 9, 2019).

19 See Paulo J. Lisboa and Azzam F. G. Taktak, *The Use of Artificial Neural Networks in Decision Support in Cancer: A Systematic Review*, 19 NeuralNetw. 408, 415 (2006).

20 AI techniques have also been developed for diagnosis and prognosis of stroke with more accuracy than existing prediction methods *id.* Jiang et al., *supra* note 13.

21 An example of this was published in the Annals of Oncology in 2018 in which a DL system (trained on over 100,000 skin images for diagnosing malignant melanomas) outperformed a group of trained physicians *id.* V. J. Mar and H. P. Soyer, *Artificial Intelligence for Melanoma Diagnosis: How Can We Deliver on the Promise?* 29 Ann. Oncol. 1625, 1628 (2018).

22 See C. Castaneda et al., *Clinical Decision Support Systems for Improving Diagnostic Accuracy and Achieving Precision Medicine*, 5 J. Clin. Bioinforma. 4 (2015).

23 See Z. Obermeyer and Ezekiel J. Emanuel, *Predicting the Future—Big Data, Machine Learning, and Clinical Medicine*, 375 N. Eng. J. Med. 1216, 1219 (2016).

24 See W. Glauser, *Artificial Intelligence, Automation and the Future of Nursing*, CanadianNurse (2017), https://www.canadian-nurse.com/en/articles/issues/2017/may-june-2017/artificial-intelligence-automation-and-the-future-of-nursing (accessed Jan. 10, 2019).

25 See D. Muoio, *Japan Is Running Out of People to Take Care of The Elderly, So It’s Making Robots Instead Business Insider* (2015), https://www.businessinsider.com/japan-developing-carebots-for-elderly-care-2015-11 (accessed Jan. 9, 2019).

26 See A. Sharafoddini, Joel A. Dubin and J. Lee, *Patient Similarity in Prediction Models Based on Health Data: A Scoping Review*, 5 JMIR Med. Inform. e7 (2017).

27 ‘*Precision in the context of public health has been described as improving the ability to prevent disease, promote health, and reduce health disparities in populations by (1) applying emerging methods and technologies for measuring disease, pathogens, exposures, behaviors, and susceptibility in populations; and (2) developing policies and targeted implementation programs to improve health’ id*. Muin J. Khoury and S. Galea, *Will Precision Medicine Improve Population Health?*, 316 JAMA 1357, 1358 (2016); See also S. Dolley, *Big Data’s Role in Precision Public Health*, 6 Front. PublicHealth, 2 (2018), https://www.ncbi.nlm.nih.gov/pmc/articles/PMC5859342/ (accessed Jan. 24, 2019).

28 See eg Ruth R. Faden et al., *An Ethics Framework for a Learning Health Care System: A Departure from Traditional Research Ethics and Clinical Ethics*, 43 HastingsCenterReport S16, S27 (2013).

29 L. Olsen, D. Aisner and J. Micheal McGinnis, *The Learning Healthcare System: Workshop Summary* 6 (2007).

30 See Tom L. Beauchamp and Y. Saghai, *The Historical Foundations of the Research-Practice Distinction in Bioethics*, 33 Theor. Med. Bioeth. 45, 56 (2012); See also G. Bertier, A. Cambon-Thomsen and Y. Joly, *Is it Research or Is It Clinical? Revisiting an Old Frontier Through the Lens of Next-Generation Sequencing Technologies*, 61 Eur. J. Med. Genet. 634, 641 (2018).

31 See Faden et al., *supra* note 29 at 23.

32 See O. Diggelmann and M. Nicole Cleis, *How the Right to Privacy Became a Human Right*, 14 Hum. RightsLawRev. 441, 458, 442 (2014).

33 See J. Halpert, J. Kashatus and K. Lucente, *Data Protection Laws of the World: The United States* (2017), https://www.dlapiperdataprotection.com/index.html?c=US&c2=&go-button=GO&t=law (accessed Jan. 17, 2019).

34 A. Levin and M. Jo Nicholson, *Privacy Law in the United States, the EU and Canada: The Allure of the Middle Ground*, 2 U. Ottawa L. & Tech. J. 357, 396, 357 (2005).

35 See P. Bender, *The Canadian Charter of Rights and Freedoms and the United*, 28 McGill L. J. 56, 820 (1983).

36 See S. Armstrong, *What Happens to Data Gathered by Health and Wellness Apps?* 353 BMJ 353 (2016).

37 As revealed by the New York Times in 2012, the store retailer Target was able to detect, based on her purchase habits, if a woman was pregnant and her approximate due-date, in order to send promotional coupons at the opportunate time. See C. Duhigg, *How Companies Learn Your Secrets*, TheNewYorkTimes (Feb. 16, 2012), https://www.nytimes.com/2012/02/19/magazine/shopping-habits.html (accessed Jan. 10, 2019).

38 O. Solon and O. Laughland, *Cambridge Analytica closing after Facebook data harvesting scandal The Guardian* (2018), https://www.theguardian.com/uk-news/2018/may/02/cambridge-analytica-closing-down-after-facebook-row-reports-say (accessed Mar. 13, 2019).

39 See European Commission, *The GDPR: New Opportunities, New Obligations* (2018), https://ec.europa.eu/commission/sites/beta-political/files/data-protection-factsheet-sme-obligations_en.pdf (accessed Feb. 6, 2019).

40 Regulation (EU) 2018/1807 of the European Parliament and of the Council of November 14, 2018 on a framework for the free flow of non-personal data in the EU. [2018] OJ L119/1.

41 See L. Pangrazio and N. Selwyn, *‘Personal Data Literacies’: A Critical Literacies Approach to Enhancing Understandings of Personal Digital Data*, 21 NewMed. & Soc. 419, 437 (2019).

42 See D. Thoren-Peden and C. Meyer, *Data Protection 2018 International Comparative Legal Guides*, in DataProtection 2018 | Laws andRegulations | USA | ICLG (2018), http://iclg.com/practice-areas/data-protection-laws-and-regulations/usa (accessed Feb. 7, 2019).

43 Adopting restrictive and disparate conditions for the processing of sensitive data could create intra-EU conflicts of law between Member States, which may eventually hinder cross-border research, especially in the field of genetics. See K. Pormeister, *Genetic Research and Applicable Law: The Intra-EU Conflict of Laws as a Regulatory Challenge to Cross-Border Genetic Research*, J. LawBiosci. (2019), https://academic.oup.com/jlb/advance-article/doi/10.1093/jlb/lsy023/5172932 (accessed Jan. 3, 2019).

44 Examples of PHI include demographic data, medical histories, test results, insurance information, and other information used to identify a patient or provide healthcare services or healthcare coverage, see What is Protected Health Information? HIPAA Journal (2018), https://www.hipaajournal.com/what-is-protected-health-information/ (accessed Jan. 24, 2019).

45 The Health Insurance Portability and Accountability Act of 1996 (HIPAA). P.L. No. 104-191, 110 Stat. 1938 (1996).

46 See A. Tanner, *Strengthening Protection of Patient Medical Data The Century Foundation* (2017), https://tcf.org/content/report/strengthening-protection-patient-medical-data/ (accessed Mar. 13, 2019).

47 See Nicholson W. Price and Glenn I. Cohen, *Privacy in the Age of Medical Big Data*, 25 Nat. Med. 37, 43, 39 (2019).

48 See K. Benitez and B. Malin, *Evaluating Re-identification Risks With Respect to the HIPAA Privacy Rule*, 17 J. Am. Med. Inform. Assoc. 169, 177 (2010).

49 See J. Kulynych and Henry T. Greely, *Clinical Genomics, Big Data, and Electronic Medical Records: Reconciling Patient Rights With Research When Privacy and Science Collide*, 4 J. Law. Biosci. 94, 132, 122 (2017).

50 Personal Information Protection and Electronic Documents Act (PIPEDA), SC 2000, c 5b.

51 Office of the Privacy Commissioner of Canada, Summary of privacy laws inCanada (2014), https://www.priv.gc.ca/en/privacy-topics/privacy-laws-in-canada/02_05_d_15/ (accessed Jan. 9, 2019).

52 See J. Stoddart, B. Chan and Y. Joly, *The European Union’s Adequacy Approach to Privacy and International Data Sharing in Health Research*, 44 J. LawMed. Ethics 143, 155 (2016).

53 See ‘Canada’s archaic privacy laws are not up to that task. Modern laws are urgently needed to protect us, as both citizens and consumers. (…) Trust needed to allow the digital economy to flourish hinges on having an appropriate legal framework’ see D. Therrien, *Op-ed: Facebook allegations underscore deficiencies in Canada’s privacy laws* (2018), https://www.priv.gc.ca/en/opc-news/news-and-announcements/2018/oped_180326/ (accessed Jan. 24, 2019).

54 See Recommandation 16, *Towards Privacy by Design: Review of The Personal Information Protection and Electronic Documents Act (2018),*https://www.ourcommons.ca/DocumentViewer/en/42-1/ETHI/report-12/ (accessed Mar. 13, 2019).

55
*Council of Canadian Academies, Accessing Health and Health-Related Data in Canada* 15 (2015).

56 Martin Coulter, *What is GDPR? Everything You Need to Know About the New EU Laws*, EveningStandard (2018), https://www.standard.co.uk/news/uk/what-is-gdpr-everything-you-need-to-know-about-the-new-eu-data-protection-laws-a3847396.html (accessed Jan. 16, 2019).

57 G. Chassang, *The Impact of the EU General Data Protection Regulation on Scientific Research*, 11 Ecancermedicalscience 709 (2017).

58 CNIL, Délibération SAN-2019-001 (2019).

59 ‘It is often not possible to fully identify the purpose of personal data processing for scientific research purposes at the time of data collection. Therefore, data subjects should be allowed to give their consent to certain areas of scientific research when in keeping with recognised ethical standards for scientific research.’ (Recital 33, GDPR)

60 “Article 29 Working Party—Guidelines on Consent under Regulation 2016/679”, (Apr. 10, 2018), https://ec.europa.eu/newsroom/article29/document.cfm?action=display&doc_id=51030.

61 See Chassang, *supra* note 55 at 3.

62 By allowing data subjects to easily transfer their data from one company to another (held by a health application for example), the right to data portability is thought to favor competition and encourage companies to better protect their customers’ privacy in order to retain them.

63 See A. St John, *Europe’s GDPR Brings Data Portability to U.S. Consumers*, ConsumerReports (2018), https://www.consumerreports.org/privacy/gdpr-brings-data-portability-to-us-consumers/ (accessed Jan. 9, 2019).

64 See P. Quinn, *Is the GDPR and Its Right to Data Portability a Major Enabler of Citizen Science?* 18 Global Jurist (2018), https://www.degruyter.com/view/j/gj.ahead-of-print/gj-2018-0021/gj-2018-0021.xml (accessed Jun. 19, 2019).

65 See *The National Committee on Vital and Health Statistics, Health Information Privacy Beyond HIPAA: A 2018 Environmental Scan of Major Trends and Challenges* 68 2 (2017).

66 See *Towards Privacy By Design: Review Of The Personal Information Protection And Electronic Documents Act*, *supra* note 52.

67 See Steven C. Bennett, *The Right to Be Forgotten: Reconciling EU and US Perspectives*, 30 Berkeley J. Int’l L. 161, 195, 161 (2012).

68 See D. F. Sittig et al., *New Unintended Adverse Consequences of Electronic Health Records*, YearbMedInform 7, 12 (2016).

69 Guidelines on Data Protection Impact Assessment (DPIA) and determining whether processing is “likely to result in a high risk” for the purposes of Regulation 2016/679 (2017).

70 See S. Gardner, *High-Risk Processing Triggers EU Data Reg Obligations*, Bloomberg (2016), https://%3A%2F%2Fwww.bna.com%2Fhighrisk-processing-triggers-n57982070517%2F (accessed Feb. 8, 2019).

71 A. Cavoukian et al., *Remote Home Health Care Technologies: How to Ensure Privacy? Build It In: Privacy by Design*, 3 IDIS 363, 378 (2010).

72 See Recommandation 14 in TowardsPrivacyByDesign: ReviewOfThePersonalInformationProtectionAndElectronicDocumentsAct, *supra* note 52 at 52.

73 See P. Voigt and A. Von dem Bussche, The EU GeneralDataProtectionRegulation (GDPR) 38 (Springer ed. 2017).

74 See Personal Information Protection Act, 34.1 (AB); Access To Information And Protection Of Privacy Act, 64.4 (NL); Personal Health Information Protection Act, 16.2 (ON); Personal Health Information Privacy and Access Act Subsection, 49.2 (NB).

75 See Gardner and 2016, *supra* note 64.

76 See Stacey A. Tovino, *The HIPAA Privacy Rule and the EU GDPR: Illustrative Comparisons*, 47 SetonHall L. Rev. 973, 994 (2016).

77 See Office of the Privacy Commissioner of Canada, *PIPEDA Legislation and Related Regulations* (2015), https://www.priv.gc.ca/en/privacy-topics/privacy-laws-in-canada/the-personal-information-protection-and-electronic-documents-act-pipeda/r_o_p/ (accessed Jan. 17, 2019).

78 See P. Kosseim, *Law in Canada—DLA Piper Global Data Protection Laws of the World* 15 54 (2018).

79 See Therrien, *supra* note 51.

80 See D. Simberkoff, *How Facebook’s Cambridge Analytica Scandal Impacted the Intersection of Privacy and Regulation*, CMS Wire (2018), https://www.cmswire.com/information-management/how-facebooks-cambridge-analytica-scandal-impacted-the-intersection-of-privacy-and-regulation/ (accessed Jan. 17, 2019).

81 See Stoddart, Chan, and Joly, *supra* note 50 at 152.

