# Supplementary material for: Integrating artificial intelligence into health care through data access: can the GDPR act as a beacon for policymakers?
Source: J Law Biosci. 2019 Sep 16;6(1):317–35. doi: 10.1093/jlb/lsz013 (PMC6813940; doi:10.1093/jlb/lsz013)
Supplement: Response_to_Decision_Letter_July_4_lsz013 [file response_to_decision_letter_july_4_lsz013.docx]

|  | **Reviewer 1 Comment** | **Response** |
| --- | --- | --- |
| **1.** | In the discussion of the dual purpose of the GDPR (P8, lines 15-26), the authors do not point out that data concerning health is a special category of data in the GDPR, and thus is treated special. In particular, the processing of data concerning health is prohibited with some exceptions. The aim to reduce costs and minimize administrative requirements is true, but there are especially exceptions in cases of processing on a large scale of special categories of data (e.g., Art. 35ff.). The distinction of sensitive data/other types of data (P9) should thus already be made here. The authors should also add here relevant Articles of the GDPR. | A sentence introducing the distinction between sensitive and non-sensitive data in the GDPR has been added where suggested. The exceptions brought by Article 35, have been developed further below, by expanding the section on Data Protection Assessment (as suggested in other comment). (Given the word limit, the authors however chose to avoid the repetition). |
| **2.** | The authors point out (P8, lines 29-35) that the data protection regulations in the EU, US and Canada only cover the collection and the use of personal data. However, they miss a very important legal development in the EU, namely Regulation (EU) 2018/1807 on a framework for the free flow of non-personal data in the European Union. | Lines added in part II.2 to include Regulation 2018/1807 and the distinction between personal/non-personal data in the EU. |
| **3.** | Some sentences about the territorial scope of the GDPR would be desirable. | Sentences have been added in the introduction. |
| **4.** | The direct quote of recital 156 of the GDPR is incorrect, and needs to be corrected (P13, lines 32-34). | Direct quote of Recital 156 corrected. |
| **5.** | In the discussion of the right to erasure, the authors should add one or two sentences about the exceptions of such right (Article 17(3) of the GDPR). | Exceptions and precisions added in part III.2.b). |
| **6.** | Some sentences about automated processing (Article 22 of the GDPR) would be desirable. | A paragraph about automated processing has been added in the section about reinforcing individuals’ sense of control over their data. |
|  | • P1, line 30: The United Kingdom is still a Member State of the European Union; depending on the time of publication, I would consider rewriting this sentence (e.g., “(…) European Commission and the United Kingdom”). | Correction made (“and the United Kindgom” deleted). |
|  | • P1, line 42: I would delete the words “in 2016” because they could be misleading. The GDPR entered into force on May 24, 2016, but only became directly applicable in all Member States two years later on May 25, 2018 (Art. 99 of the GDPR). | Words “in 2016” deleted. |
|  | • P2, line 10: The authors should also cite Alan Turing’s paper “Computing Machinery and Intelligence”, available at: https://www.csee.umbc.edu/courses/471/papers/turing.pdf. | Reference added. |
|  | • P2, line 14: It would be nice to add one or two sentences about what has changed since 1959. Why is AI nowadays so promising? | The authors acknowledge that such addition is interesting but took the liberty to avoid lengthening the section on the description of AI (given the restricting word limit and the fact that this addition was considered minor by the reviewer). |
|  | • P2, line 30: The hyphen between “United Kingdom” should be deleted. | Hyphen deleted. |
|  | • P3, line 8: The hyphen between “data sharing” should be deleted for consistency. | Hyphen deleted. |
|  | • P4, lines 9 and 10: The sentence would be clearer as “the term “artificial intelligence” refers to …”; the words “was coined in 1956” should be deleted since they are not relevant for the definition. If the authors want to keep such words, they should clarify in an additional sentence that the term was coined in 1956 by John McCarthy and colleagues. | Sentence has been changed and the date deleted. |
|  | • P4, line 19: The example of Watson as a machine-learning system is in so far not ideal since this section deals with the successful deployment of AI in healthcare. However, Watson for Oncology has come under criticism recently for often providing incorrect treatment recommendations. This should at least be mentioned in the footnote. | The example has been deleted. |
|  | • P7, line 39: The word “is” needs to be deleted. | The word “is” has been added. |
|  | • P9, line 6: The word “new European regulation on data protection” should be replaced by “GDPR.” | The word “new European regulation on data protection” has been replaced by “GDPR.” |
|  | • P10, lines 13, 14: A relevant paper in this area is Price II/Cohen, privacy in the age of medical big data, Nature Medicine. | Reference added. |
|  | • P11, line 28: The words “deeming PIPEDA” need to be deleted. | The words “deeming PIPEDA” have been deleted. |
|  | • P15, line 21 and 36: I would insert a new paragraph for the US and Canadian framework. | A new paragraph has been inserted. |
|  | • The footnotes are currently inconsistent (some are before, some after the point). | All footnotes before the point have been put after and are now consistent. |

|  | **Reviewer 2 Comment** | **Response** |
| --- | --- | --- |
| **1.** | As discussed by the authors on pages 7 and 8, some interpretations suggest EU law creates a distinction between data protection and privacy law. Privacy law is designed to protect intrusion into privacy space. By contrast, data protection law is more specifically concerned with ‘informational self-determination’. Accordingly, restructure the sentence as follows:  *Through the adoption of its new General Data Protection Regulation (GDPR), the EU was the first in attempting to regulate AI through data protection legislation. This regulation paved the way for meaningful reforms in privacy legislation in the US and Canada.* | Sentence restructured as suggested. |
| **2.** | It should be noted that PIPEDA has already been certified as adequate under the GDPR by the European Commission subject to Article 45. However, this adequacy determination extends to private organizations only. | Paragraph has been reformulated to explain how PIPEDA is currently benefitting from the exception set by Article 46 GDPR which allows adequacy status granted under the Directive to remain valid until further revision by the European Commission. |
| **3.** | I do not necessarily agree with this reading of the exceptions under the GDPR. My assessment is that under Articles 6(1)(a) and 9(2)(a), specific informed consent is the default ground for the processing of personal data. However, that does not necessarily dispense with the requirement to obtain some form of consent before processing data. Potential guidance in this regard comes from an opinion issued by the EDPB in January 2019. This opinion related to the conflict between the requirement for consent under the GDPR and the new Clinical Trials Regulation (CTR). Notably, the CTR does not mandate a requirement of specific, informed consent, but instead requires written, free consent for participation in a clinical trial. The EDPB confirmed that the appropriate legal basis for data processing relating to reliability and safety purposes under the CTR is Article 6(1)(c). However, reliance on Article 6(1)(c) does not obliviate the requirement to rely on consent under the CTR.  *Interestingly, these exceptions are established as alternative conditions. The wording of the article thus implies that obtaining specific, informed consent as required under the GDPR is not necessary, as long as another legal basis for the processing applies.* | Sentence restructured as suggested. |
| **4.** | The authors are correct in noting that Recital 33 does recognise the difficulty in obtaining specific consent for scientific research. However, Recital 33 has been contradicted by other statements from the European Data Protection Board and the former Article 29 Data Protection Working Party. Both of these bodies have significant interpretative power under the GDPR. The Article 29 Working Party Guidelines on consent under Regulation 2016/679 (adopted 28 November 2017) explicitly addressed consent for scientific research under Recital 33. The Article 29 Working Party noted that Recital 33 did not dispense with the requirement for specific consent. Instead, scientific research projects can include personal data where that data has been obtained with the consent of data subjects. Where a research project cannot be described with sufficient specificity, then the project may be described in more general terms. Nevertheless, the Article 29 Working Party held that GDPR does not permit a controller to navigate around the key principle of specifying consent.  Incorporating this discussion into the article would require fixing the sections on informed consent. It might be possible for the authors to link this discussion to the sentence on dynamic consent. In particular, the Article 29 Working Party raised dynamic consent on a case by case basis as an alternative to where specific consent could not be obtained. | The paragraph on informed consent has been fixed to include the recommendations of the Article 29 Working party. |
| **5.** | With respect to this issue, a useful article that the authors may wish to refer to is Paul Quinn, ‘Is the GDPR and Its Right to Data Portability a Major Enabler of Citizen Science?’, Global Jurist, 18(2) (2018). Quinn establishes that the right to data portability has a number of limitations. Importantly, data can only be transferred where it is personal data and supplied by the data subject. As alluded to by the authors, this requirement is designed as a balance on the competitiveness requirement imposed by the data portability requirement. In other words, the data portability right does not require a data processor or controller to relinquish control over valuable trade secrets for processing data. Accordingly, for personal health data, the right of data portability only provides a mechanism for transferring personal health related data. It does not extend to the analyses of this data. | Suggested reference added and a limit to Article 20 has been developed accordingly. |
| **6.** | First, it should be noted that the GDPR sets high sanctions for the breach of all provisions (including the data processing principles). The right to erasure should not be taken as a unique case in this regard. Secondly, the right of erasure is linked to a number of exceptions where it may not apply. For example, Article 17(1)(a) implicitly permits an exception to the right of erasure where the personal data is necessary for processing for the purpose for which the data was collected. In other words, a data processor or controller may be able to refuse a deletion request if they can demonstrate the data is required for processing. Whilst Article 17(1)(b) also permits deletion where data was collected on the grounds of consent and the subject withdraws that consent, there may be other exceptions to refuse erasure. | - Modification of the sentence to make clear that high sanctions apply for the violation of all provisions of the GDPR, including the right to erasure.  - Suggested exception to the right to erasure has been added. |
| **7.** | As stated above, the right of erasure is qualified by a number of exceptions. These exceptions are contained in Article 17(3) of the GDPR. First, Article 17(1) shall not apply to the extent that data is to be processed to comply with Union or state law for the public interest. In the alternative, data may not need to be erased where the processing involves the exercise of official authority (Article 17(3)(b)). Secondly, data may not be erased where processed for reasons of public interest in the area of public health (Article 17(3)(c)). Both of these provisions may be relied upon by a hospital to avoid deleting data.  Also, how might this right of erasure affect the use of AI in healthcare? The previous section on data portability contained a summary of the relevant legislative requirements in the EU, the US and Canada. It then proceeded to provide an example of how this right might allow individuals to control their data. What effect might the right of erasure have on the use of AI? A possible conclusion might relate the authors’ previous assertion that DL and ML leads to data and software being inextricably linked. Is it possible that this linking might increase the difficulty in exercising the right of erasure? I would elaborate on this section in further detail.  Finally, what are the risks of erasure on the integrity of patient records? It would be useful to have a source here demonstrating what the potential risks of incompatibility in health records are. | - Exceptions to the right to erasure have been added.  - A possible difficulty raised by DL algorithm to the application of the right to erasure has been added.  - Existing problems regarding the lack of consistency in electronic health records have been explained and linked to the right to erasure. |
| **8.** | It should be noted that although Article 35 does provide specific examples of high-risk processing where a DPIA is needed. First, systematic and extensive evaluation based on automated processing that produces legal or other significant effects for natural persons may be high risk (Article 35(3)(a)). Secondly, processing on a large scale of special categories of personal data or personal data relating to criminal convictions or offences may constitute high risk processing (Article 35(3)(b)). Thirdly, a systematic monitoring of a public area may constitute high risk processing (Article 35(3)(c)). However, it should be noted that the use of the words in particular suggests that this list is non- exhaustive.  Accordingly, the Article 29 Working Party issued Guidelines on Data Protection Impact Assessment (DPIA) and determining whether processing is ‘likely to result in a high risk’ in 4 October 2017. The following nine criteria must be considered:  1) Evaluation or scoring, including profiling and predicting;  2) Automated decision making with legal or similar significant effects; 3) Systematic monitoring and processing used to observe, monitor and control data subjects, including data collected through networks;  4) Sensitive data or data of a highly personal nature, including data about individual political opinions, patient records or private information;  5) Data processed on a large scale (either via population size, volume of data, duration of processing, or the geographical extent of processing);  6) Matching or combining datasets, particularly where it would exceed the reasonable expectations of the data subject; 7) Data concerning vulnerable data subjects (including children, employees, mentally ill persons, asylum seekers, the elderly, patients) or where there is an imbalance between the data subject and the controller;  8) Innovative use of existing technology, or applying new technology or organisational solutions;  9) Where the process in of itself prevents data subjects from exercising a right or using a service or contract.  Given these exceptions, the section on data protection impact assessments should be significantly expanded. In addition, a conclusion on the effect of a DPIA on the use of AI should be drawn here. It may be possible to conclude that given the Article 29 Working Party’s pronouncements, all data processing via AI should be subject to a DPIA given the presence of principle 8. However, as an addendum, the authors should acknowledge that the need for a DPIA depends on the data being processed. | Sentences to explain to clarify what a “high-risk” processing under the GDPR means and exceptions have been added. The suggested conclusion was thought very relevant and used in the paragraph. |
| **9.** | If this sentence is the start of a new paragraph, create an additional space and indent the first sentence | New paragraph created. |
| **10.** | Reword this sentence in an active voice:  ‘In the United Kingdom, citizens rejected the care.data project launched in 2014 due to privacy concerns.’ Also, one of the key concerns about care.data was the lack of an obvious mechanism to allow patients to opt out of having their records uploaded. I would specify these concerns in more detail. In particular, this question of opt out consent is directly related to the requirement of specific consent under the GDPR. More needs to be made of it in the article. | Sentence reworded as suggested. More details on the care.data have been added. |
| **11.** | Split this sentence into two.  Paradoxically, this lack of trust is counterbalanced by a growing popularity of direct-to-consumer genetic testing and health monitoring devices. These devices create massive flows of personal and health data, mostly to private companies. | Sentence splitted in two as suggested. |
| **12.** | I would rephrase this sentence  *Deep learning describes a more complicated form of ML reliant on artificial neural networks.*  *Convoluted is not the right adjective to use in this sentence.* | Sentence reworded as suggested. |
| **13.** | Split this sentence in two:  *As of today, AI in healthcare is generally concentrated around three fields; oncology, neurology and cardiology. These are all areas of medicine in which early detection is crucial.* | Sentence splitted in two as suggested. |
| **14.** | Split this sentence in two:  *The blurring of the boundary between research and practice entailed by the LHS does come in conflict with traditional legal and ethical norms. These norms are built around the belief that research and clinical care need to be clearly delineated to protect patients and research participants.* | Sentence splitted in two as suggested. |
| **15.** | Fix the typo in this sentence. Also, ‘this’ lacks a noun. What is concerning? Is it ‘individual user habits’, or is it the ability to surmise health data from non-health related data?  For example:  *These contradictory habits can be concerning, since health data is often logically surmised from other personal data provided online, such as consumption habits.* | Typo fixed and sentence reworded as suggested. |
| **16.** | Change to:  *They also highlight the lack of adapted privacy and governance frameworks to prevent unwanted use of personal data, especially on the Internet.* | Change made. |
| **17.** | Split sentence in three. Also, change which is a subtype a deidentification process as follows:  *Strict conditions are set around anonymization under the GDPR. Anonymization involves a number of techniques designed to prevent reidentification by ‘[taking account of] all the means reasonably likely to be used, such as singling out, either by the controller or by another person to identify the natural person directly or indirectly.’*  Also, conclude by changing Recital 26 into a quote. | Sentence changed as suggested and Recital 26 has been put into a quote. |
| **18.** | Delete the extra deeming PIPEDA  It should also be noted that adequacy determinations are issued by the European Commission, and not the Article 29 Working Party/European Data Protection Board. | Extra “deeming PIPEDA” deleted and clarification on the institution has been made. |
| **19.** | Change as follows:  *However, in both frameworks, what constitutes valid consent is not always in line with the GDPR’s new dispositions, especially as to the point of explicitness.* | Change made. |
| **20.** | Change as follows:  *Specifically, the GDPR intends to prevent implied consent scenarios, such as ‘opt-out based participation’. In these scenarios, it is not always clear to data subjects what they are actually consenting to.* | Change made. |
| **21.** | Change as follows:  *Including new requirements such as privacy by design and by default in privacy law would substantially help in reducing the incidence of data breaches in the US and Canada.* | Change made. |
| **22.** | Change as follows:  *This simplification comes at the cost of granting national privacy authorities higher investigatory and sanction powers.* | Change made. |
| **23.** | Change as follows:  *A certain level of reluctance from the public is understandable given the high-profile examples of misuses of personal data such as revealed by the Cambridge Analytica case.* | Change made. |
|  | Overall, I think the article is well written, but I am slightly confused by the final thesis proposed. It appears the article suggests that the GDPR provides a way forward for amending North American privacy laws to provide greater protection against infringements via artificial intelligence. However, if that is the thesis of the article, I would prefer **more discussion as to the merits and shortcomings of the GDPR, particularly with respect to artificial intelligence.** At present, the discussion of the operation of the GDPR and its application to artificial intelligence is not quite as nuanced as it could be. In particular, I believe the authors should elaborate on the right of erasure and the right to data portability. I would also prefer it if the authors included a **discussion on the right of automated profiling**, which is very pertinent to artificial intelligence but is narrowly construed. If these shortcomings were included, it would support the final conclusion that the GDPR could be used as grounds for reforming US and Canadian privacy law and guiding policymaking. | The conclusion has been complemented in order to address these shortcomings and clarify the general contentions of the authors. A paragraph about automated processing has also been added in Part III 1. a). |

**Substantial additional modification:**

1) Missing title for Part III: “Improving privacy and data protection in Canada and the US: insights from the GDPR” has been added.

2) Title of Part III.1.a) has been reworded as “Right to consent and Automated processing”.
